# Ubiquitination site preferences in anaphase promoting complex/cyclosome (APC/C) substrates

**DOI:** 10.1098/rsob.130097

**Published:** 2013-09

**Authors:** Mingwei Min, Ugo Mayor, Catherine Lindon

**Affiliations:** 1 Department of Genetics, University of Cambridge, Downing Street, Cambridge CB2 3EH, UK; 2 CIC bioGUNE, Bizkaia Technology Park, Building 801-A, Derio 48160, Spain; 3 IKERBASQUE, Basque Foundation for Science, 48011 Bilbao, Spain

**Keywords:** ubiquitination, ubiquitin acceptor, (APC/C), KEN, degron, Aurora A

## Abstract

Ordered progression of mitosis requires precise control in abundance of mitotic regulators. The anaphase promoting complex/cyclosome (APC/C) ubiquitin ligase plays a key role by directing ubiquitin-mediated destruction of targets in a temporally and spatially defined manner. Specificity in APC/C targeting is conferred through recognition of substrate D-box and KEN degrons, while the specificity of ubiquitination sites, as another possible regulated dimension, has not yet been explored. Here, we present the first analysis of ubiquitination sites in the APC/C substrate ubiquitome. We show that KEN is a preferred ubiquitin acceptor in APC/C substrates and that acceptor sites are enriched in predicted disordered regions and flanked by serine residues. Our experimental data confirm a role for the KEN lysine as an ubiquitin acceptor contributing to substrate destruction during mitotic progression. Using Aurora A and Nek2 kinases as examples, we show that phosphorylation on the flanking serine residue could directly regulate ubiquitination and subsequent degradation of substrates. We propose a novel layer of regulation in substrate ubiquitination, via phosphorylation adjacent to the KEN motif, in APC/C-mediated targeting.

## 2. Introduction

Faithful cell division requires a robust and efficient network to regulate the precise ordering of events. Cyclin-dependent kinases (CDKs) act as the hub of this network, with help from other major mitotic kinases such as polo-like kinases (Plks) and Aurora kinases [1]. They use phosphorylation as a common language to distinctively modulate each event. Once the cells reach metaphase, these mitotic regulators are irreversibly inactivated by targeted protein degradation. This is mediated by the ubiquitin-proteasome system, where the substrates are modified with ubiquitin chains that recruit them to the 26S proteasome [2,3].

The process of ubiquitination involves a three-enzyme cascade, where a free ubiquitin is first charged by an E1 activating enzyme and subsequently transferred to an E2 conjugating enzyme. In the presence of an E3 ligating enzyme, which binds both the E2 and the substrate, the ubiquitin is finally used to modify a substrate lysine residue. Importantly, one ubiquitin can modify a second ubiquitin molecule, resulting in eight polyubiquitin chain types with distinct structural topologies and cellular functions [4]. In rapid and tightly controlled processes like mitotic exit, therefore, these enzymes have two critical issues to solve: the specificity in substrate recognition that controls the timing of substrate ubiquitination and the specificity in building the ubiquitin chain that decides the efficiency and outcome of ubiquitination. It is generally held that E3s are likely to determine the first specificity and the E2s are the crucial factors for the second specificity [4,5].

Surprisingly, among more than 600 E3s in human cells, the anaphase promoting complex/cyclosome (APC/C) is responsible for all the known degradation events controlling mitotic exit [3,6,7]. APC/C has more than 70 reported substrates, and the total number can be estimated as at least double this [7]. Despite its wide targeting, the APC/C processes its substrates for ubiquitination in a precisely controlled sequential way [8]. APC/C substrate targeting depends on characteristic ‘degron’ motifs present in substrates, most commonly D-boxes (RxxL) and KEN motifs [9,10]. However, degron recognition alone cannot account for the specificity and efficiency of substrate degradation. Where studied in detail, substrates are generally found to contain multiple and partially redundant degrons that are thought to bind to core subunits of the APC/C and to associated specificity factors Cdc20 or Cdh1 [11]. Indeed, the most recent crystallographic structure study reveals multiple binding sites on the Cdh1 surface that interact with different degrons and cryo-EM studies indicate a composite receptor for D-boxes between Cdh1 and APC10 [12,13].

The APC/C uses two specific E2 enzymes for substrate ubiquitination, UBE2C for initial conjugation of one (‘priming’) ubiquitin molecule to substrates and UBE2S to build a polyubiquitin chain on this ubiquitin molecule [14–16]. UBE2S specifically catalyses K11-linked polyubiquitin chain formation by binding the TEK-box of ubiquitin through a surface close to its active site [17]. For ‘priming’ of substrate lysines, an initiation motif has been proposed to promote ubiquitination efficiency by providing a favoured E2 interacting surface in APC/C substrates [17]. By contrast, little is known about preferences for particular substrate lysines, possibly because these surfaces are dynamically regulated in the cell, for example, by other post-translational modifications (PTMs) that cannot be easily recapitulated in *in vitro* systems.

Several recent mass spectrometry-based ubiquitome studies have identified large numbers of *in vivo* ubiquitination sites [18–25]. This has been facilitated by the increased data that have become available through use of ‘diGly capture proteomics’ using a specific antibody for direct purification of the di-glycine remnant of ubiquitinated lysines following trypsin digestion [25]. Here, we clustered these ubiquitome datasets and focused on the features of identified ubiquitination sites from APC/C substrates. We report that the KEN box can be ubiquitinated in APC/C substrates. Serine residues feature strongly in both upstream and downstream flanking regions of ubiquitination sites in APC/C substrates, with particularly significant enrichment in the –1 flanking position. Using Aurora A and Nek2A as model substrates, we test the idea that phosphorylation on flanking serine residues could directly regulate substrate ubiquitination levels. Our data are consistent with novel cross-regulation between these two PTMs.

## 3. Results

### 3.1. A non-redundant compendium of ubiquitination sites in human cells

**Figure 1.**
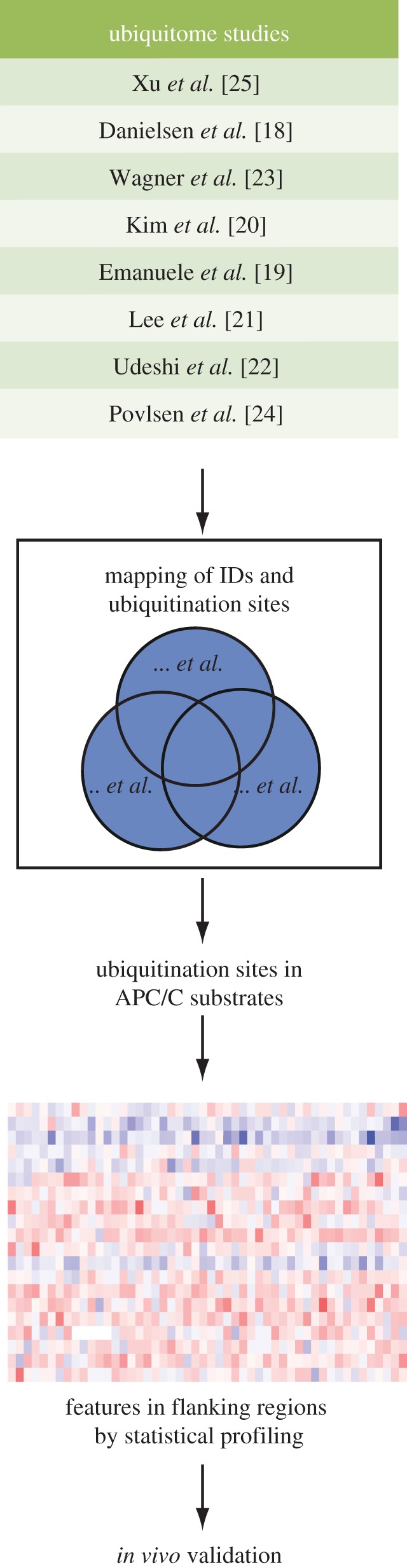
Summary of ubiquitination site datasets and strategy used in this study. Statistical analysis of flanking regions of ubiquitination sites in APC/C substrates revealed ubiquitination site preferences that were then validated *in vivo*.

We set out to identify ubiquitination motifs in APC/C substrates by compiling large-scale *in vivo* ubiquitome datasets from several previous studies (figure 1) [18–25]. All of these ubiquitination sites were experimentally identified by mass spectrometry, which detects the mass shift generated by the di-glycine remnant signature of ubiquitinated lysine after trypsin digestion [26]. When we compiled 30 609 ubiquitination sites across 7248 proteins (see electronic supplementary material, table S1) from these datasets, we observed that while most of the proteins have only a few ubiquitination sites, some of them have far more ubiquitination sites than the expectation from a random distribution. The number of proteins with a given number of ubiquitination sites follows a power law (see electronic supplementary material, figure S1), allowing us to infer that the compendium is representative of the distribution of ubiquitination sites across cellular networks, since power law (scale-free) distributions are characteristic of cellular system components [27]. Indeed, we observe the same scale-free distribution for the most studied PTM, phosphorylation (see electronic supplementary material, figure S1), as previously described [28]. Within our compendium of ubiquitination sites, 11 325 sites (approx. 37%) were identified in at least two studies (see electronic supplementary material, figure S1).

A list of experimentally verified APC/C substrates was curated from the literature (see electronic supplementary material, table S2). We extracted all the ubiquitination sites of APC/C substrates from the non-redundant compendium (see figure 1 and electronic supplementary material, table S1). It resulted in 285 ubiquitination sites from 53 APC/C substrates. Although ubiquitination on these sites may not all depend on the APC/C, we reasoned that APC/C-dependent ubiquitination sites would still be enriched in this dataset compared with the whole human ubiquitome. Thus, the statistical features present in this dataset would reflect preferences in selection of substrate lysines for ubiquitination by the APC/C, while underestimating the statistical significance of any such preferences.

### 3.2. Ubiquitination sites in APC/C substrates are enriched in predicted disordered regions

**Figure 2.**
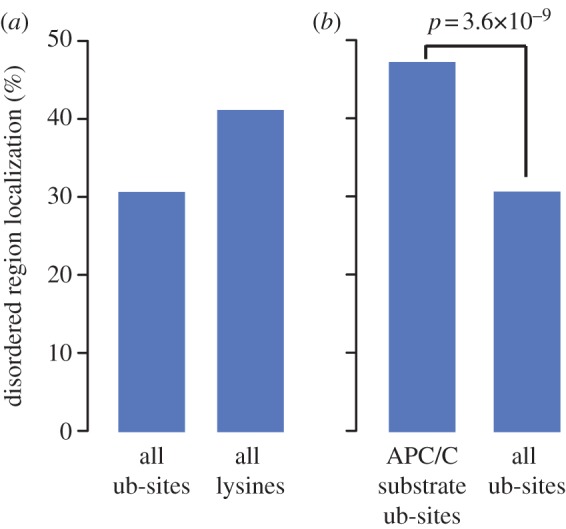
Comparison of proportion of ubiquitination sites (ub-sites) located in disordered regions. Ubiquitinated lysines in general are not enriched in disordered regions (*a*). Ubiquitination sites identified in APC/C substrates are significantly more likely to present in disordered regions than other ubiquitinated lysines (*b*). *p*-value evaluated using a binomial test.

Previous studies of APC/C substrates have shown that degrons are often found in generally unstructured regions (reviewed in [5]). We therefore tested whether the ubiquitination sites in APC/C substrates tend to reside in disordered regions, by predicting the disordered regions in all the proteins of our non-redundant compendium using DISOPRED [29]. In agreement with previous observations, globally ubiquitination sites were more frequently present in structured regions and depleted in predicted disordered regions (figure 2*a*) [18,23,25]. In contrast to these studies of all known ubiquitination sites, the ubiquitination sites in APC/C substrates were significantly enriched in disordered regions (*p*-value = 3.6 × 10^–9^, binomial test; figure 2*b*).

As the ubiquitin ligase that is responsible for all the known targeted proteolysis in mitotic exit, APC/C has to bind different substrates with high specificity, and yet with considerable flexibility to allow efficient elongation of the ubiquitin chain. The dynamic properties of disordered regions make them good structural candidates for mediating this process, and may explain the enrichment of ubiquitination sites in APC/C substrate disordered regions. Alternatively, our finding may reflect the requirement for an unfolded region in substrate proteins to initiate proteolysis at the proteasome (reviewed in [30]). Finally, our result indicates the possibility that APC/C substrate processing could be influenced by crosstalk of other PTMs with ubiquitination within disordered regions [20,31], as disordered regions tend to harbour ‘hot spots’ of PTMs such as phosphorylation [32]. Indeed, phosphorylation sites located close to modified lysines are more likely to be conserved [31], and the phosphorylation status of sites in these regions tends to vary through the cell cycle [33,34]. Therefore, the enrichment of APC/C substrate ubiquitination sites in disordered regions would be consistent with dynamic regulation of those sites.

### 3.3. Ubiquitination sites in APC/C substrates show flanking region preferences

**Figure 3.**
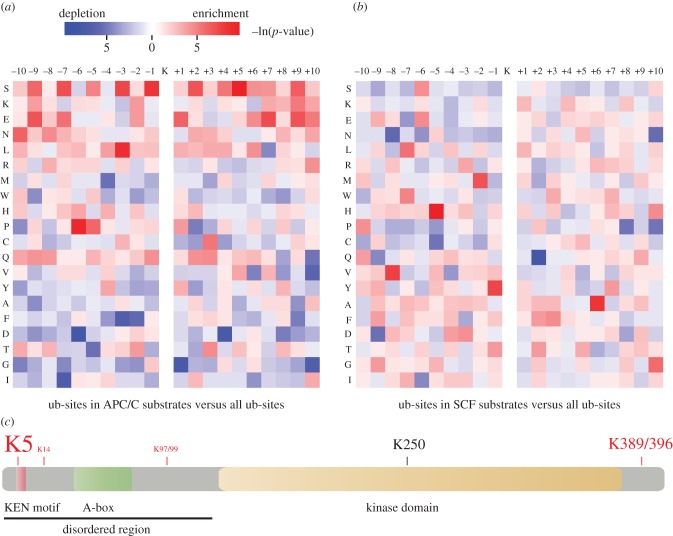
Ubiquitination site preferences in APC/C substrates. (*a,b*) Flanking region profiles of the ubiquitination sites in substrates of the APC/C (*a*) and SCF (*b*). Sites from APC/C substrates are listed in the electronic supplementary material, table S1. Sites from SCF substrates are derived from study [35]. Heat map (ln of the *p*-value) depicts significance of enrichment (in red) or depletion (in blue) for each amino acid within ±10 residues of APC/C or SCF substrate ubiquitination sites (ub-site), against all ubiquitination sites. The *p*-values were evaluated using a binomial test. (*c*) Schematic of Aurora A ubiquitination sites identified by mass spectrometry confirms preference for KEN ubiquitination. Sites identified in this study are in red, with font size reflecting the ratio of ubiquitinated : non-ubiquitinated versions of each peptide. See electronic supplementary material, figure S3 for further details.

We set out to investigate whether there might be common features around the ubiquitinated lysines of APC/C substrates that could function to promote ubiquitination or proteolysis. Different ubiquitome studies have shown that compared with all lysines in the human proteome, ubiquitination sites tend to locate in non-basic environments, although no specific motif was detected [20,23]. However, considering that different E2/E3 modules contributing to the human ubiquitome could show distinct patterns of preference for lysines targeted by ubiquitination, we tested flanking region preferences of ubiquitinated lysines in APC/C substrates (see electronic supplementary material, table S1) versus all ubiquitination sites, presenting this analysis as a heatmap of preferences within the 21-amino acid sequence centred on the modified lysine (figure 3*a*). This analysis revealed distinct preferences for certain residues in the flanking regions of APC/C ubiquitination sites. The strongest preference was for serine residues in the entire flanking region (figure 3*a*), suggesting that phosphorylation on these residues could interplay with APC/C-dependent ubiquitination. Indeed, 28% of these serines have been experimentally identified as phosphorylation sites [36], a significantly higher percentage than that of all serines in these proteins (*p*-value = 8 × 10^–4^, binomial test). This enrichment in flanking serines was most significant at the −1 position immediately flanking the ubiquitinated lysine residue (see also electronic supplementary material, figure S2). The general preference for lysines in APC/C ubiquitination site flanking regions is consistent with the observation that APC/C substrates are frequently ubiquitinated on multiple sites in the vicinity of degrons [37]. Of particular note in the heatmap of flanking region preferences was the emergence of K_0_EN as a preferred ubiquitin chain acceptor in APC/C substrates (figure 3*a*). This preference was even stronger if we refined the ubiquitination sites in APC/C substrates to those within 40 residues downstream of potential degrons (‘RxxL’ and ‘KEN’ motifs; electronic supplementary material, figure S2*b*), where APC/C-dependent ubiquitination sites are more likely to be found, based on the known spatial layout of the substrate-recognition catalytic module [12]. None of the features that we found in the ubiquitination site flanking regions of APC/C substrates was present in a heatmap of ubiquitination site flanking regions of known substrates of the SCF ubiquitin ligase complex [35] generated from the same compendium of ubiquitination sites (figure 3*b*).

### 3.4. SKEN can be a ubiquitin acceptor motif

To experimentally confirm the ubiquitination site flanking features (S_(?P)_-K_0_-E-N) that we had identified, we first searched for APC/C substrates containing an SKEN motif, to examine whether the motif can function as a ubiquitin chain acceptor *in vivo*. Eight validated APC/C substrates from our list (see electronic supplementary material, table S2) contain SKEN, three of these (in Cdc20, Nek2A and ECT2) being known sites of ubiquitination (see electronic supplementary material, table S1). We selected two substrates, hsAurora A and hsNek2A kinases, for *in vivo* study. We have previously shown that Aurora A is specifically targeted by APC/C^Cdh1^ for degradation in mitotic exit to promote assembly of a robust spindle midzone [38]. Nek2A is a substrate of APC/C-Cdc20 that is targeted for degradation in prometaphase [39]. Neither of these substrates is thought to depend on its KEN motif for targeting to the APC/C [39–43].

We first examined whether SK_5_EN is an ubiquitination site in Aurora A during mitotic exit. Only one ubiquitination site on Aurora A, K250 [19], was present in our compendium of ubiquitination sites. In order to identify further sites of ubiquitination on Aurora A, we overexpressed His_6_-tagged protein in human U2OS cells, using Aurora A *Δ*32–66 (lacking the putative ‘A-box’ degron) [41], a version of Aurora A that we have found to be present in APC/C^Cdh1^-dependent polyubiquitin conjugates at far higher levels than the wild-type protein (that is, stabilized post-ubiquitination: M.M., S. Qiao, C.L., 2010, unpublished data). We purified His_6_-Aurora A *Δ*32–66 from cells synchronized at mitotic exit when Aurora A is maximally ubiquitinated [6] and sent this material for interrogation by mass spectrometry for the presence of ubiquitinated peptides. Even using this version of Aurora A, the number of ubiquitinated peptides identified was very low. However, we identified six ubiquitinated lysines, with overall sequence coverage of 72%. The KEN lysine, K5, was the most frequently identified site of ubiquitination (figure 3*c* and see electronic supplementary material, figure S3 for details). We concluded that SK_5_EN is an important site of mitotic ubiquitination in Aurora A.

### 3.5. SK_5_EN contributes to mitotic exit-specific ubiquitination and degradation of Aurora A

**Figure 4.**
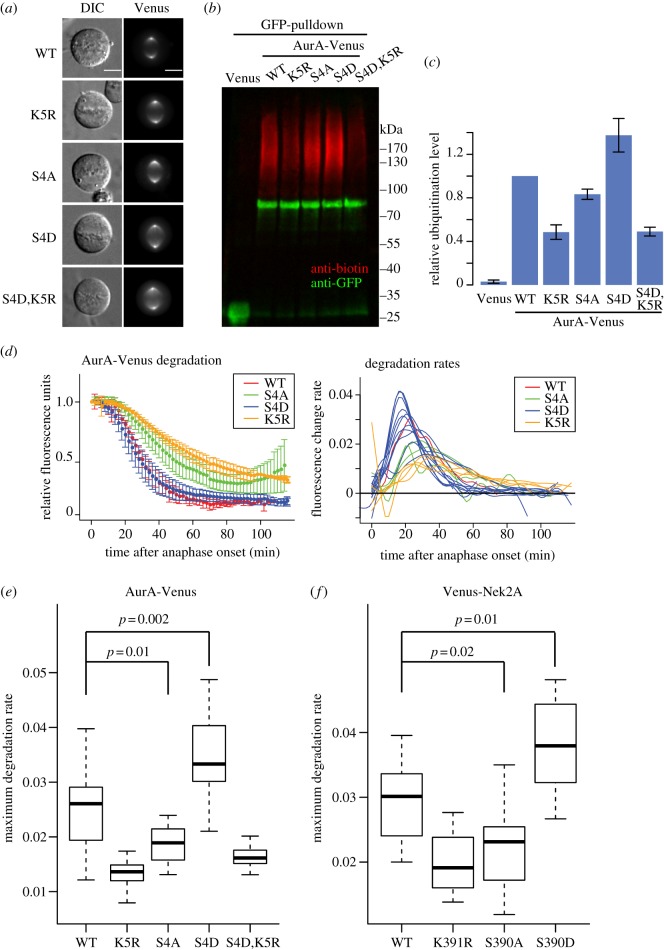
Experimental validation of the SKEN motif as a potential phospho-regulated ubiquitination site. (*a*) AurA-Venus SKEN mutants used in this study all localize like the wild-type protein at mitosis. U2OS cells expressing indicated AurA-Venus constructs were imaged in mitosis in preparation for time-lapse degradation assay described in (*d*). Bars 10 μm. (*b*) *In vivo* ubiquitination of Aurora A SKEN mutants. Biotinylated–ubiquitin-expressing U2OS cells were electroporated with indicated AurA-Venus construct for 48 h, synchronized in prometaphase by sequential thymidine/STLC block, collected by mitotic shake-off and forced to exit mitosis using ZM447439 treatment. Whole cell lysate was applied to GFP-Trap agarose beads to pull down Venus-tagged protein. Immunoblotted samples were then probed with anti-GFP and anti-biotin antibodies to detect unmodified and ubiquitinated AurA-Venus, respectively. (*c*) Quantitation of *in vivo* ubiquitination. Immunoblots from three repeats of the experiment shown in (*b*) were used to calculate the ratio of biotin signal to GFP signal for each AurA-Venus construct. The ratios were normalized to that of AurA-WT-Venus and plotted as means with standard deviations (±s.d.). (*d*) Degradation of AurA-Venus SKEN motif mutants during mitotic exit. U2OS cells expressing indicated AurA-Venus constructs were filmed by time-lapse fluorescence microscopy at 2 min intervals during mitotic exit. The fluorescence of individual cells was measured and normalized to anaphase onset, with the mean ± s.d. from at least five cells plotted for each protein (left-hand panel). The degradation rates of AurA-Venus in individual cells were calculated and plotted using an in-house R script (right-hand panel). (*e,f*) Maximum proteolysis rates of SKEN-dependent APC/C substrates are sensitive to phosphorylation status of the motif. (*e*) Distribution boxplot of maximum degradation rates of Aurora A SKEN motif mutants from figure 4*d. P*-values were determined using Student's *t*-test. (*f*) Distribution boxplot of maximum degradation rates of Nek2A SKEN motif mutants shown in the electronic supplementary material, figure S5.

To test the contribution of SK_5_EN to ubiquitination and degradation of wild-type Aurora A, we substituted the lysine for arginine (K5R) in full-length Aurora A. Venus-tagged Aurora A-K5R showed identical localization to wild-type Aurora A-Venus (figure 4*a*). To compare the ubiquitination capacity of these two proteins *in vivo*, we expressed them in a human U2OS cell line [44] also expressing biotin-tagged ubiquitin [45] and synchronized the cells in mitotic exit. Taking advantage of the affinity purification reagent GFP-Trap, that we have found to bind GFP derivatives even under denaturing conditions, we purified Aurora A-Venus from these synchronized cells. Use of denaturing conditions stabilizes ubiquitin-conjugated material, allowing us to purify both unmodified and ubiquitinated Venus-tagged proteins in this assay. We found that after blotting of purified material, we could quantify the unmodified and ubiquitinated fractions using anti-GFP and anti-biotin antibodies, respectively. The high sensitivity of the biotin antibody allows reproducible quantification of very small amounts of polyubiquitinated material. We measured the ratio of polyubiquitinated to unmodified Aurora A in this assay and found that ubiquitination on AurA-K5R-Venus was reduced compared with AurA-WT-Venus (figure 4*b,c*). We confirmed that the K5-dependent ubiquitination of Aurora A contributes to its mitotic destruction by measuring Aurora A proteolysis during time-lapse imaging of live cells [38]. Quantification of fluorescence levels in single cells showed that AurA-K5R-Venus was degraded at a significantly lower rate than AurA-WT-Venus (figure 4*d*). Taken together, our data suggest that K5 in the SKEN motif is a major ubiquitination site mediating Aurora A degradation during mitotic exit. We cannot exclude that the KEN motif also contributes to Aurora A ubiquitination through its potential function as a degron, Indeed we have found that the triply substituted KEN > AAA shows a reduced rate of degradation during mitotic exit compared with the version where the lysine residue alone is substituted, K5 > R (see electronic supplementary material, figure S4*b*). It seems likely therefore that the KEN motif plays a dual role in binding to the APC/C and accepting ubiquitin during Aurora A degradation. We tested the doubly substituted KEN > KAA in order to examine further whether K5 plays a more significant role as a ubiquitin acceptor or as part of a degron, but this version of Aurora A (unlike KEN > AAA) did not behave like the wild-type protein in mitotic cells (see electronic supplementary material, figure S4*a*) and was not suitable for further analysis.

### 3.6. Phospho-regulation of ubiquitination efficiency at SKEN motifs

A large number of proteins are highly phosphorylated at mitosis, and phosphate groups on modified serines in disordered regions could provide a negatively charged environment to promote the efficiency of ubiquitination of APC/C substrates [46]. This argument would appear to hold in particular for the serine residue at the −1 position, which we identified as highly enriched in the list of ubiquitinated peptides from APC/C substrates (see figure 3*a* and electronic supplementary material, figure S2). As serine S4 is a known phosphorylation site on Aurora A *in vivo* [47], we examined whether ubiquitination on K5 might be influenced by the phosphorylation status of S_4_KEN. We found that the phospho-mimetic version AurA-S4D-Venus showed increased ubiquitination, as well as a higher maximum degradation rate in mitotic exit, compared with AurA-WT-Venus (figure 4*b–e*). Conversely, the non-phosphorylable version AurA-S4A-Venus showed reduced ubiquitination and a reduced rate of degradation during mitotic exit. The increased ubiquitination and degradation rate of AurA-S4D was fully reversed by additional mutation of K5 (AurA-S4D,K5R-Venus, figure 4*b*,*c*,*e*). Together, these results suggest that the phosphorylation status of S4 would regulate the ubiquitination efficiency, or the level of ubiquitin occupancy, on K5 of Aurora A.

We tested whether the SK_391_EN motif of Nek2A could mediate a similar response to phosphorylation status of the –1 serine. Nek2A degradation depends on its carboxy-terminal methionine–arginine dipeptide tail to mediate binding to APC/C core subunits during spindle checkpoint (SAC)-independent degradation at prometaphase, in combination with its dimerization via a leucine zipper [39,40]. A number of ubiquitome studies have identified Nek2A lysine K391 as a ubiquitination site *in vivo* [19,20]. Consistent with previous findings that mutating its KEN box does not change the timing of degradation *in vivo* [40], we found that Venus-Nek2A-K391R was degraded during prometaphase with similar timing to Venus-Nek2A-WT (see electronic supplementary material, figure S5). However, in line with what we had found for AurA-K5R-Venus, the maximum rate of degradation was reduced (figure 4*f*). Meanwhile, the maximum degradation rate was significantly increased by a phospho-mimetic substitution S390D and reduced by non-phosphorylable S390A (figure 4*f*). In agreement with our observations made with Aurora A, phosphorylation of SKEN can likely act to promote ubiquitination at K391 of Nek2A. Nek2A S390 is not a listed phosphorylation site in existing phospho-proteome studies, but is a potential site for CK1 phosphorylation (ELM database of eukaryotic linear motifs [48]).

In summary, we validated our finding that APC/C substrates show sequence preferences in the flanking regions of ubiquitin acceptor sites, by demonstrating that the SKEN motif provides such a site, where the occupancy of the acceptor could be promoted by phosphorylation on the –1 serine residue.

## 4. Discussion

In this study, we have found specific preferences in ubiquitination site selection in APC/C substrates, with particular preferences for disordered regions and those enriched in serine residues. We have furthermore demonstrated that the KEN box can be ubiquitinated *in vivo* and that this ubiquitination event may be regulated at least partly by phosphorylation on flanking serine.

Our identification of APC/C-dependent ubiquitination preferences is necessarily limited firstly because it is not possible to know which sites identified by mass spectrometry in known APC/C substrates are in fact APC/C-dependent, and secondly because APC/C-dependent ubiquitination events leading to rapid degradation of those substrates might be underrepresented in mass spectrometry studies compared with ubiquitination events leading to slower/no degradation. Nonetheless, we were able to extract statistically significant preferences, which could be reinforced by refining our analysis to ubiquitination sites close to APC/C degron motifs, and that we subsequently confirmed *in vivo*.

Our finding that the KEN motif can be ubiquitinated seems at first sight curious given the well-described role of KEN as an APC/C-specific degron motif. It raises the possibility that the degron function of the KEN motif could be regulated by ubiquitination. Moreover, it has repeatedly been found that inhibition of the APC/C by pseudo-substrates critically depends on KEN motifs [49–53], consistent with the idea that they would act to bind the APC/C in the absence of significant ubiquitination of the pseudo-substrate. Indeed, for at least one pseudo-substrate, Acm1, it has been shown that the KEN lysine alone cannot support polyubiquitination in the absence of neighbour lysines [50]. However, the observation that weakening the interaction between Acm1 and APC/C leads to increased ubiquitination (and degradation) of Acm1 [50,51] is consistent with models for substrate ubiquitination where dissociation of substrate from E2/E3 is a necessary step in the addition of post-priming ubiquitins to the chain. In this scenario, ubiquitination of the KEN motif could contribute to the complex control of APC/C-substrate affinity via multiple degrons that determines the fate of potential substrates. It will be important to develop quantitative *in vivo* assays for binding of APC/C to its substrates in order to test the relative contributions of KEN binding and ubiquitination to substrate degradation *in vivo*. Nonetheless, our data suggest that the contribution of the KEN motif to substrate degradation is more complex than existing models allow for.

From our finding that serines are preferred in the flanking region of APC/C ubiquitination sites we hypothesized that APC/C substrates would show phosphorylation-mediated regulation of their ubiquitination. Indeed, there are several known examples where APC/C-mediated substrate degradation is regulated by phosphorylation, including that of key substrates securin, Aurora A and Cdc6 [54–56]. However, in each of these examples, phosphorylation was shown to inhibit the accessibility of degrons to the APC/C, thus playing a negative role in substrate targeting for proteolysis. By contrast, our experiments predict phosphorylation at sites flanking ubiquitin acceptor lysines to play a positive role in substrate ubiquitination, most probably downstream of degron binding by the APC/C.

The transfer of ubiquitin from E2 to substrate requires nucleophilic activation of a substrate lysine for attack on the E2-ubiquitin thioester bond. Much of our insight into the mechanism of catalysis comes from studies of lysine modification with SUMO, an ubiquitin-like modifier. Ubc9, an E2 for SUMOylation, provides a hydrophobic microenvironment in its active site that desolvates the acceptor lysine to lower its effective p*K_a_*, enhancing deprotonation. This promotes nucleophilic attack by acceptor lysine on the thioester bond at physiological pH [46]. By the same reasoning, the flanking regions of substrate lysines should also favour a negatively charged environment. Indeed, this has been shown to be statistically true for the whole human ubiquitome [20,31] and provides a rationale for our observed enrichment for glutamic acids in APC/C substrate ubiquitination site flanking regions, in particular at the +1 position where it could be predicted to contribute to efficient modification on K_0_EN. A large number of proteins are highly phosphorylated at mitosis, and phosphate groups on modified serines in disordered regions could provide a negatively charged environment to promote the efficiency of ubiquitination of APC/C substrates. Pathways controlling SKEN phosphorylation status have not yet been described. Nonetheless, the particular enrichment of serine over threonine in our analysis suggests that these phosphorylation events could be highly specific.

Together, our experiments show that S_(P)_-K-E-N could behave as an efficient motif for ubiquitin chain modifications in promoting mitotic degradation of both Aurora A and Nek2A, supporting our computational analysis showing preferential ubiquitination on lysines flanked by phosphorylatable serines in APC/C substrates. This is the first time it has been shown that the APC/C shows preferences for ubiquitin acceptor sites in any specific sequence context, and that this preference could influence ubiquitination efficiency on APC/C substrates downstream of degron recognition. This preference is likely to contribute to the many dimensions of regulation that control polyubiquitinated substrates ready for mitotic destruction.

## 5. Material and methods

### 5.1. Ubiquitination site datasets

Ubiquitination site data were obtained from existing studies [18–25]. Protein IPI identifiers were converted to UniProt ID using the ID mapping file on the UniProt FTP server. Ubiquitination sites were verified against FASTA sequences from UniProt database. The datasets were merged and this procedure resulted in a compendium containing a total of 30 609 unique ubiquitination sites that could be mapped to 7428 unique UniProt protein identifiers.

### 5.2. Disordered region and flanking region analyses

The intrinsic disordered state was predicted for each ubiquitination site using DISOPRED with standard settings [29]. Using the binomial test, we tested the null hypothesis that the frequency of observing ubiquitination sites in disordered regions is not greater in APC/C substrates than in all ubiquitinated proteins.

To identify amino acids that were enriched or depleted in the flanking regions of ubiquitination sites, we extracted ±10 residues of each site in the compendium. For each amino acid at each position relative to the ubiquitination site, we tested the null hypothesis that the frequency of observing this amino acid is not greater (for enrichment analysis) or not less (for depletion analysis) in the APC/C substrates than in all ubiquitinated proteins, against the alternative hypothesis that it is. *p*-values from binomial tests were ln-transformed and plotted as a heat map using the R environment [57] and the gplots package [58]. Statistical analyses were performed using the R environment [57].

### 5.3. Plasmids

His_6_-tagged Aurora A*Δ*32–66 was expressed using pcDNA3.1(+)/myc-His C vector (Invitrogen).

Venus was swapped for fluorescent proteins in pEYP-N1-AurA [38] and pEGFP-C1-Nek2A [39] (kind gift of Prof. Andrew Fry) to make pVenus-N1-AurA and pVenus-C1-Nek2A.

K5R, S4A, S4D, S4D,K5R versions of pVenus-N1-AurA and K391R, S390A, S390D versions of pVenus-C1-Nek2A were generated using standard mutagenesis techniques. Full cloning details available upon request.

### 5.4. Cell culture and synchronization

U2OS cells (parental) or U2OS tet-OFF cells expressing biotinylatable ubiquitin [44] were cultured in DMEM supplemented with 10% FBS, antibiotics and amphotericin B (all from PAA Laboratories, Pasching, Austria). U2OS tet-OFF cell culture medium was additionally supplemented with 1 μg ml^−1^ tetracycline hydrochloride (Calbiochem, San Diego, CA, USA). Expression of Venus-tagged constructs was achieved by electroporation using Invitrogen Neon system according to the manufacturers' instructions. Cells were analysed 12–48 h following electroporation.

Synchronization in mitotic exit was achieved using SAC-dependent mitotic arrest by kinesin-5 inhibitor S-trityl-l-cysteine (STLC) treatment for 16 h. Cells were collected by mitotic shake-off and forced into mitotic exit by silencing the SAC using Aurora B inhibitor ZM447439 for 70 min.

### 5.5. His-tag protein purification

For each sample, about 10^8^ cells were lysed in 10 ml lysis buffer (8 M urea, 1% (v/v) SDS, 1X protease inhibitor cocktail (EDTA-free, from Roche) and 50 mM *N*-ethylmaleimide in 1× PBS). The lysate was applied to 450 µl beads (Dynabeads His-Tag Isolation & Pulldown from Life Technologies) and incubated for 1 h at room temperature. The beads were then washed twice with wash buffer 1 (lysis buffer + 200 mM NaCl, pH 6.3) followed by once with wash buffer 2 (wash buffer 1 + 10 mM imidazole). Three sequential elutions were performed by incubating the beads with 1 ml elution buffer (wash buffer 1 + 10 mM EDTA, pH 4.3) for 30 min at room temperature. The eluate was pooled together and concentrated using a Vivaspin Centrifugal Concentrator.

### 5.6. Mass spectrometry

Samples were processed by the Cambridge Centre For Proteomics. MS/MS spectra were searched using the Mascot search engine (Matrix Science) against a Swissprot database restricted to human entries, with a maximum of three missed cleavages allowed. Carbamidomethylation was set as fixed modification while methionine oxidation and GlyGly modification on K were included as variable modifications. The search was performed with an initial mass tolerance of 1 Da for the precursor ion and 0.6 Da for the MS/MS spectra. Error-tolerant search was allowed to identify other potential modifications.

### 5.7. *In vivo* ubiquitination assays

Biotin–ubiquitin cells were transfected with indicated AurA-Venus construct for 48 h, and synchronized to SAC-dependent mitotic arrest using STLC, collected by mitotic shake-off and forced into mitotic exit by silencing the SAC using Aurora B inhibitor ZM447439 for 70 min. For each sample, about 5 × 10^6^ cells were lysed in 100 µl lysis buffer (50 mM Tris–HCl pH 7.6, 150 mM NaCl, 1 mM EDTA, 0.5% (v/v) triton; 1×protease inhibitor cocktail (Roche); 1 mM NaF; 1 mM Na_3_VO_4_; 50 mM *N*-ethylmaleimide). Following dilution with 400 µl dilution buffer (10 mM Tris–HCl pH 7.6, 150 mM NaCl, 0.5 mM EDTA, 1×protease inhibitor cocktail; 1 mM NaF, 1 mM Na_3_VO_4_, 50 mM *N*-ethylmaleimide), the lysate was applied to 5 µl GFP-Trap_A beads and incubated for 2 h at room temperature. The beads were then washed with dilution buffer followed by thrice with 1 ml wash buffer 1 (8 M urea + 1% (v/v) SDS in PBS) and once with wash buffer 2 (1% (v/v) SDS in PBS). To minimize the non-specific binding and the binding of Aurora A interactors, each wash with wash buffer 1 was performed on rollers for 5 min. The beads were then boiled in 5 μl elution buffer (4 × Laemmli buffer; 100 mM DTT) for 10 min at 95°C and the eluted sample was separated from the beads by centrifugation. Blots were probed with GFP antibody (ab290 from Abcam, 1 : 2 K; secondary IRDye 800CW-conjugated goat anti-rabbit from LI-COR, 1 : 15 K) and biotin antibody (1 : 100, no. 7075 from Cell Signaling, HRP-conjugated), for quantitative detection of unmodified and ubiquitinated AurA-Venus, respectively, using a LI-COR Odyssey machine.

### 5.8. *In vivo* degradation assays

U2OS cells were electroporated with AurA-Venus or Venus-Nek2A constructs and seeded at 2 × 10^4^ cm^−2^ onto eight-well plastic-bottom slides (Ibidi GmbH, Martinsried, Germany) for time-lapse analyses. Imaging medium was L-15 supplemented with FBS and antibiotics. Time-lapse imaging was carried out on an Olympus CellR imaging platform comprised of Olympus IX81 motorized inverted microscope, Orca CCD camera (Hamamatsu Photonics, Japan), motorized stage (Prior Scientific, Cambridge, UK) and 37°C incubation chamber (Solent Scientific, Segensworth, UK). Epifluorescent and DIC images were acquired with 1 × 1 bin using appropriate filter sets and 40× NA 1.3 oil objective, at 2 min intervals. Image sequences were exported as 12-bit tiff files for analysis in ImageJ. Degradation curves and rate curves were plotted with an in-house script using R environment, with degradation rates calculated from the gradient of the tangent at each point of degradation curves after smoothing [57].

## Supplementary Material

Supplementary figures S1 - S5, with figure legends

## Supplementary Material

Supplementary Table 1

## Supplementary Material

Supplementary Table 2
