# Supplementary material for: Ubiquitination site preferences in anaphase promoting complex/cyclosome (APC/C) substrates
Source: Open Biol. 2013 Sep;3(9):130097. doi: 10.1098/rsob.130097 (PMC3787748; doi:10.1098/rsob.130097)

Supplementary figure legends

Figure S1

Statistics of ubiquitination sites in the non-redundant compendium compiled in electronic supplementary material, table S1. (***a***-***b***) Breakdown of ubiquitinated proteins by the number of modified sites per protein (***a***) compared to that of phosphorylation sites (***b***) from the human proteome. (***c***) Breakdown of ubiquitination sites by the number of studies that identified them. 37% of sites fall in categories 2-8 (plotted here on log scale).

Figure S2

(***a***) Multiple testing correction of flanking region preferences. To apply the most rigorous test possible to the statistical significance of the identified amino acid enrichments in the flanking region of APC/C ubiquitination sites, we applied a multiple testing correction to the calculated *p*-values ($p\ [corrected] = 1 - (1 - p)^n$). This test recalculates confidence measures allowing for random occurrence of the identified enrichment at any particular position within multiple positions [1]. This correction would underestimate the significance of enrichment at individual positions, but even so the *p*-value for serine at -1 position remained significant.

(***b***) Ubiquitination sites close to potential degrons show stronger preference for serine at -1 and in 'KEN' motif than general ubiquitination sites in APC/C substrates. Right-hand panel: Flanking region profile of ubiquitination sites within 40 residues downstream of potential degrons ('RxxL' and 'KEN' motifs) in APC/C substrates. Heat map (ln of the *p*-value) depicts significance of enrichment (in red) or depletion (in blue) for each amino acid as described in figure 2a-b. Left-hand panel is adopted from figure 2a for comparison.

Figure S3

Identification of ubiquitination sites in Aurora A by mass spectrometry.

(***a***) Sequence coverage achieved is shaded yellow, with the A-box in red. The number of ubiquitinated peptides identified was very low (***b***), reflecting our general observation that <1% of total substrate is

polyubiquitinated at any one time. We used Aurora AΔA-box rather than the full-length protein, because we can achieve much higher levels of expression of this version, since this protein is still ubiquitinated in APC/C-dependent manner, but not efficiently degraded (MM, S. Qiao, CL, unpublished data).

Figure S4

Further investigation of the Aurora A KEN motif. (***a***) U2OS cells expressing indicated AurA-Venus constructs were imaged in mitosis in preparation for time-lapse degradation assays. AurA-Venus KEN>KAA did not localise correctly at mitosis, even though AurA-Venus KEN>AAA localised exactly like the wild-type protein. Bars, 10 μm. (***b***) *In vivo* degradation assays (performed as described in figure 4d) for different AurA-Venus KEN mutants showed that AurA-Venus KEN>KAA is completely stable during mitotic exit and that AurA-Venus KEN>AAA is partially degraded, although more slowly than the wild-type protein or AurA-Venus KEN>REN. We concluded that Aurora A KEN probably contributes to both degron and ubiquitin acceptor functions in Aurora A degradation but that we could not test this idea further using Aurora A KEN>KAA since this version of Aurora A (mislocalized, likely misfolded) was not suitable for further study.

Figure S5.

In vivo degradation assay for Nek2A WT and SKEN mutants. Time-lapse imaging and quantifications were performed as in figure 4d. NEBD, Nuclear Envelope Breakdown.

1. Noble WS (2009) How does multiple testing correction work? *Nature Biotechnology* **27:** 1135-1137

Supplementary figure S1

(*a*)

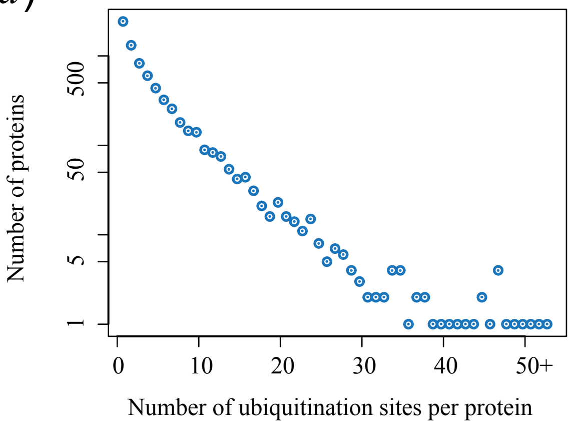

(*b*)

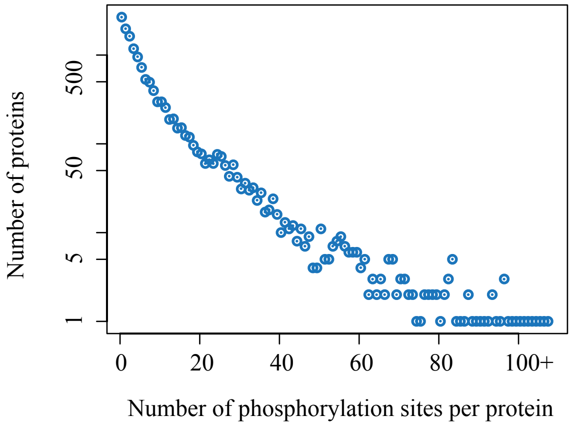

(*c*)

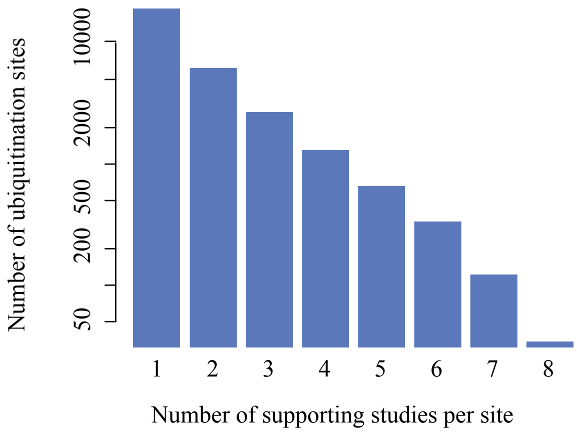

Supplementary figure S2

(*a*)

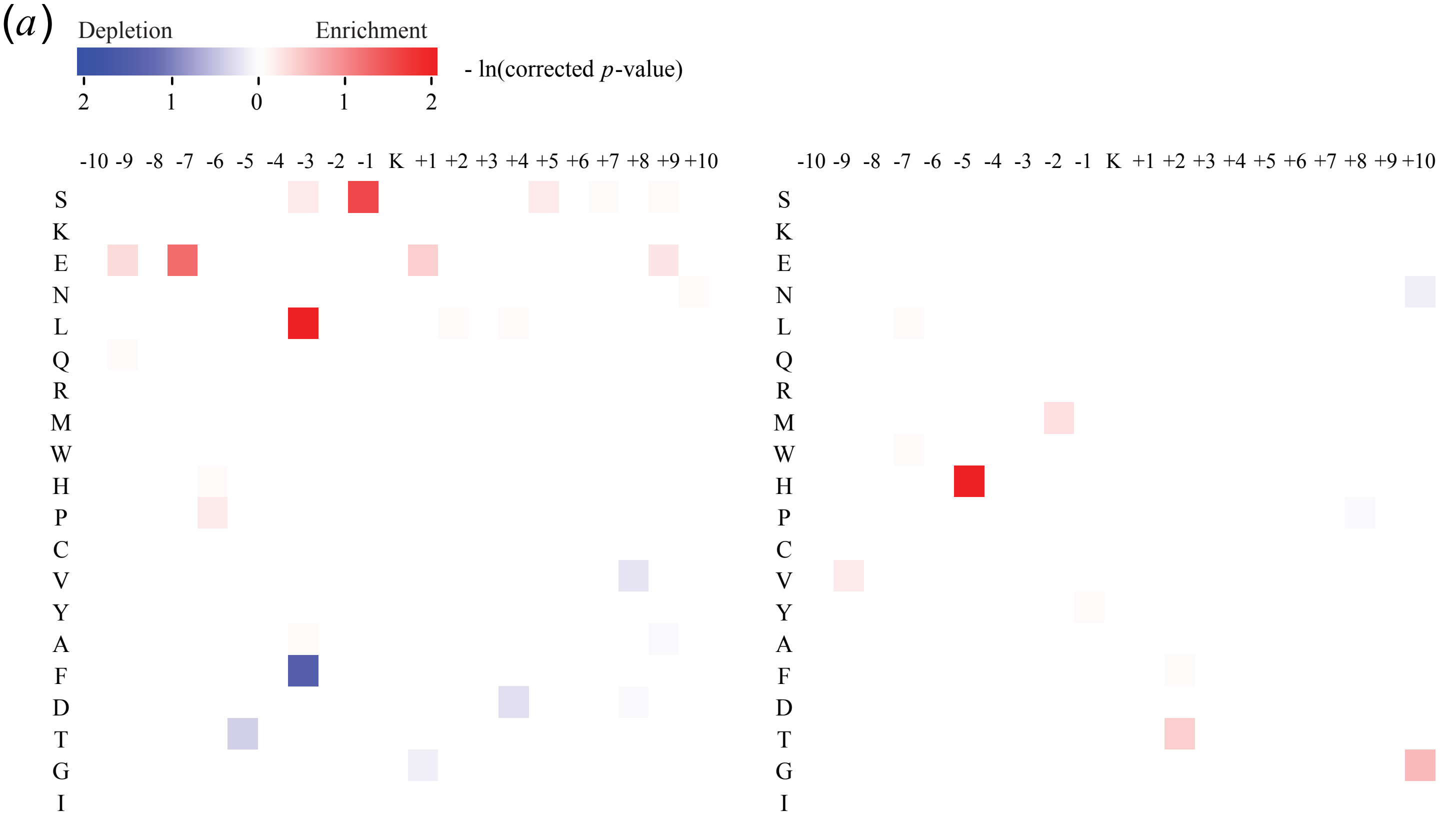

ub-sites in APC/C substrates vs all ub-sites

ub-sites in SCF substrates vs all ub-sites

(*b*)

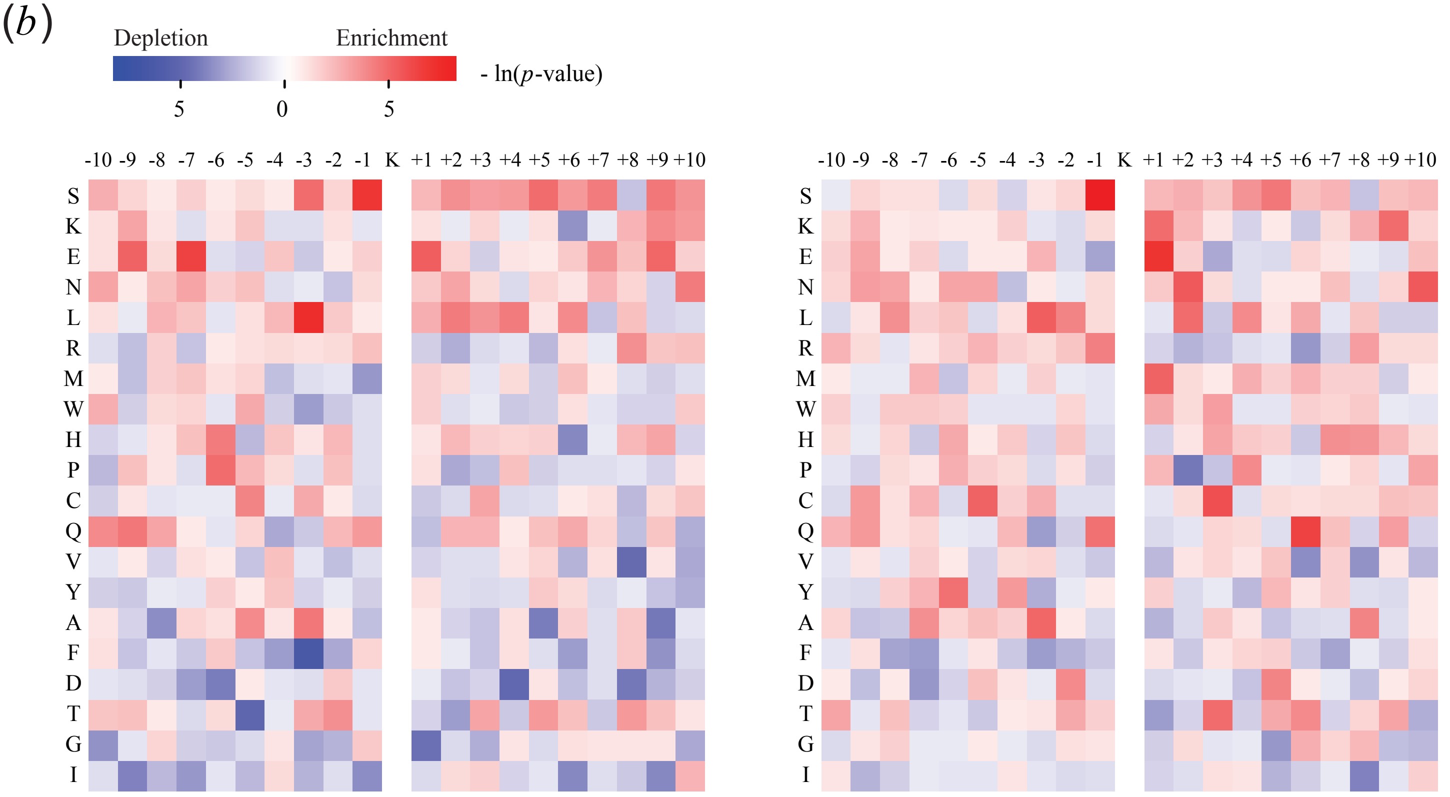

ub-sites in APC/C substrates vs all ub-sites

ub-sites in APC/C substrates ≤ 40 residues downstream of D-box or KEN motif vs all ub-sites

Supplementary figure S3

(*a*)

```
        10         20         30         40         50         60         70         80         90
MDRSKENCISGPVKATAPVGGPKRVLVTQQFPCQNPLPVNSGQAQRVLCPSNSSQRVPLQAQKLVSSHKPVQNQKQKQLQATSVPHPVSR
       100        110        120        130        140        150        160        170        180
PLNNTQKSKQPLPSAPENNPEEELASKQKNEESKKRQWALEDFEIGRPLGKGKFGNVYLAREKQSKFILALKVLFKAQLEKAGVEHQLRR
       190        200        210        220        230        240        250        260        270
EVEIQSHLRHPNILRLYGYFHDATRVYLILEYAPLGTVYRELQKLSKFDEQRTATYITELANALSYCHSKRVIHRDIKPENLLLGSAGEL
       280        290        300        310        320        330        340        350        360
KIADFGWSVHAPSSRRTTLCGTLDYLPPEMIEGRMHDEKVDLWSLGVLCYEFLVGKPPFEANTYQETYKRISRVEFTFPDFVTEGARDLI
       370        380        390        400
SRLLKHNPSQRPMLREVLEHPWITANSSKPSNCQNKESASKQS
```

72% coverage of whole Aurora A sequence
82% coverage of Aurora A sequence excluding the unidentifiable peptides in deleted A-box

(*b*)

Ubiquitination sites	Unmodified peptide no.	Ubiquitinated peptide no.
K5	14	3
K14	17	1
K97/99	14	1
K389/396	7	1

Supplementary figure S4

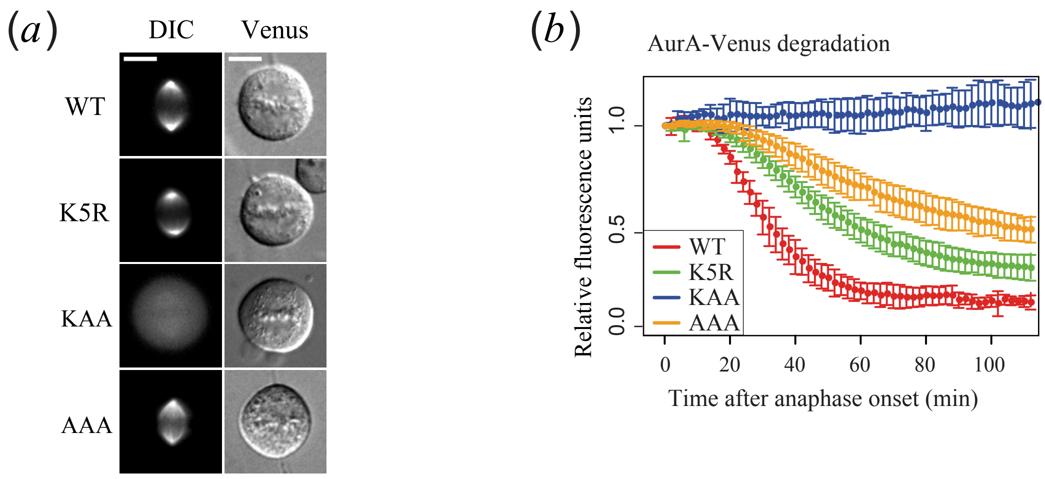

Supplementary figure S5

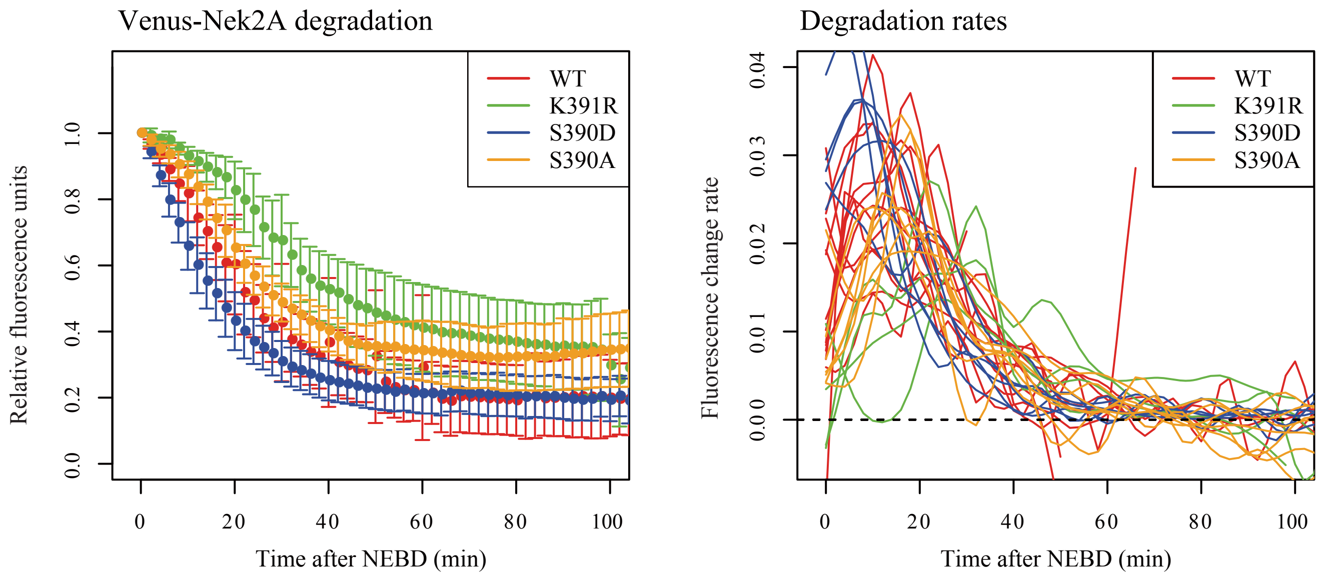

Supplement: Supplementary figures S1 - S5, with figure legends [file rsob130097supp1.pdf]
