# Supplementary material for: Ubiquitination site preferences in anaphase promoting complex/cyclosome (APC/C) substrates
Source: Open Biol. 2013 Sep;3(9):130097. doi: 10.1098/rsob.130097 (PMC3787748; doi:10.1098/rsob.130097)
Supplement: Supplementary Table 2 [file rsob130097supp3.docx]

Supplementary Table 2

| Substrate | UniProt ID | Gene name |  |
| --- | --- | --- | --- |
| **Taken from Meyer & Rape 2011** | | | |
| Anillin | Q9NQW6 | ANLN |  |
| Aurora A | O14965 | AURKA |  |
| Aurora B | Q96GD4 | AURKB |  |
| B99 | Q9NYZ3 | GTSE1 |  |
| BARD1 | Q99728 | BARD1 |  |
| Bub1 | O43683 | BUB1 |  |
| Cdc6 | Q99741 | CDC6 |  |
| Cdc20 | Q12834 | CDC20 |  |
| Cdh1 | Q9UM11 | FZR |  |
| CDR2 | Q01850 | CDR2 |  |
| CENPF | P49454 | CENPF |  |
| Cdc25 | P30304 | CDC25A/CDC25A |  |
| Cdt1 | Q9H211 | CDT1 |  |
| CKAP2 | Q8WWK9 | CKAP2 |  |
| Cks1 | P61024 | CKS1B |  |
| Claspin | Q9HAW4 | CLSPN |  |
| Cyclin A | P20248 | CCNA2 |  |
| Cyclin B | P14635 | CCNB1 |  |
| E2-C | O00762 | UBE2C |  |
| FoxM1 | Q08050 | FoxM1 |  |
| Geminin | O75496 | GMNN |  |
| glutaminase 1 | O94925 | GLS |  |
| Hec1 | O14777 | NDC80 |  |
| Hmmr | O75330 | HMMR |  |
| HSF2 | Q03933 | HSF2 |  |
| HURP | Q15398 | DLGAP5 |  |
| Id2 | Q02363 | ID2 |  |
| JNK1 | P45983 | MAPK8 |  |
| JNK2 | P45984 | MAPK9 |  |
| Kid | Q14807 | KIF22 |  |
| Nek2a | P51955 | NEK2 |  |
| Nlp | Q9Y2I6 | NINL |  |
| NuSAP | Q9BXS6 | NUSAP1 |  |
| p21 | P38936 | CDKN1A |  |
| Pfkfb3 | Q16875 | PFKFB3 |  |
| PIF | Q9H611 | PIF1 |  |
| Plk1 | P53350 | PLK1 |  |
| Rcs1 | Q9BSJ6 | FAM64A |  |
| Securin | Q5FBB7 | PTTG2 |  |
| Sgo1 | Q5FBB7 | SGOL1 |  |
| Skp2 | Q13309 | SKP2 |  |
| SnoN | P12757 | SKIL |  |
| sororin | Q96FF9 | CDCA5 |  |
| TK1 | P04183 | TK1 |  |
| TMPK | P23919 | DTYMP |  |
| Tome-1 | Q99618 | CDCA3 |  |
| TRB3 | Q96RU7 | TRIB3 |  |
| TPX2 | Q9ULW0 | TPX2 |  |
| PAF | Q15004 | PAF |  |
|  |  |  |  |
| **Others** |  |  | **references** |
| RASSF1A | Q9NS23 | RASSF1A | [[1](#_ENREF_1)] |
| Cyclin B3 | Q8WWL7 | CCNB3 | [[2](#_ENREF_2)] |
| p190RhoGAP | Q9NRY4 | ARHGAP35 | [[3](#_ENREF_3)] |
| Ect2 | Q9H8V3 | ECT2 | [[4](#_ENREF_4)] |
| USP1 | O94782 | USP1/USP1 | [[5](#_ENREF_5)] |
| USP37 | Q86T82 | USP37 | [[6](#_ENREF_6)] |
| Drp1 | O00429 | DNM1L | [[7](#_ENREF_7)] |
| p63 | Q9H3D4 | TP63 | [[8](#_ENREF_8)] |
| centrin | O15182 | **C**ETN3 | [[9](#_ENREF_9)] |
| E2F1 | Q01094 | E2F1 | [[10](#_ENREF_10), [11](#_ENREF_11)] |
| E2F3 | O00716 | E2F3 | [[12](#_ENREF_12)] |
| Mcl1 | Q07820 | MCL1 | [[13](#_ENREF_13)] |
| MOAP-1 | Q96BY2 | MOAP1 | [[14](#_ENREF_14)] |
| OPA1 | O60313 | OPA1 | [[15](#_ENREF_15)] |
| TFAM | Q00059 | TFAM | [[15](#_ENREF_15)] |
| MFN1 | Q8IWA4 | MFN1 | [[15](#_ENREF_15)] |
| Receptor-associated protein 80 | Q96RL1 | UIMC1 | [[16](#_ENREF_16)] |
| Mps1 | P33981 | TTK | [[17](#_ENREF_17), [18](#_ENREF_18)] |
| NIPA | Q86WB0 | ZC3HC1 | [[19](#_ENREF_19)] |
| G9a | Q96KQ7 | EHMT2 | [[20](#_ENREF_20)] |
| GLP | Q96KQ7 | EHMT1 | [[20](#_ENREF_20)] |
| Sp100 | P23497 | SP100 | [[21](#_ENREF_21)] |
| Oct1 | P14859 | POU2F1 | [[22](#_ENREF_22)] |

Substrates that contain SKEN motif identified as ubiquitin acceptor

Substrates that contain SKEN motif not identified as ubiquitin acceptor

Substrates that contain SK identified as ubiquitin acceptor

1. Chow, C., et al., *Regulation of APC/CCdc20 activity by RASSF1A-APC/CCdc20 circuitry.* Oncogene, 2011.

2. Nguyen, T.B., et al., *Characterization and Expression of Mammalian Cyclin B3, a Prepachytene Meiotic Cyclin.* Journal of Biological Chemistry, 2002. **277**(44): p. 41960-41969.

3. Naoe, H., et al., *The Anaphase-Promoting Complex/Cyclosome Activator Cdh1 Modulates Rho GTPase by Targeting p190 RhoGAP for Degradation.* Molecular and Cellular Biology, 2010. **30**(16): p. 3994-4005.

4. Liot, C., et al., *APC cdh1 Mediates Degradation of the Oncogenic Rho-GEF Ect2 after Mitosis.* PLoS ONE, 2011. **6**(8): p. e23676.

5. Cotto-Rios, X.M., et al., *APC/CCdh1-dependent proteolysis of USP1 regulates the response to UV-mediated DNA damage.* The Journal of Cell Biology, 2011. **194**(2): p. 177-186.

6. Huang, X., et al., *Deubiquitinase USP37 Is Activated by CDK2 to Antagonize APCCDH1 and Promote S Phase Entry.* Molecular Cell, 2011. **42**(4): p. 511-523.

7. Horn, S.R., et al., *Regulation of mitochondrial morphology by APC/CCdh1-mediated control of Drp1 stability.* Molecular Biology of the Cell, 2011. **22**(8): p. 1207-1216.

8. Hau, P.M., et al., *Loss of ΔNp63α promotes mitotic exit in epithelial cells.* FEBS Letters, 2011. **585**(17): p. 2720-2726.

9. Lukasiewicz, K.B., et al., *Control of Centrin Stability by Aurora A.* PLoS ONE, 2011. **6**(6): p. e21291.

10. Peart, M.J., et al., *APC/C Cdc20 targets E2F1 for degradation in prometaphase.* Cell Cycle, 2011. **9**(19): p. 3956-3964.

11. Budhavarapu, V.N., et al., *Regulation of E2F1 by APC/C<sup>Cdh1</sup> via K11 linkage-specific ubiquitin chain formation.* Cell Cycle, 2012. **11**(10): p. 2030-2038.

12. Ping, Z., et al., *APC/C<sup>Cdh1</sup> controls the proteasome-mediated degradation of E2F3 during cell cycle exit.* Cell Cycle, 2012. **11**(10): p. 1999-2005.

13. Harley, M.E., et al., *Phosphorylation of Mcl-1 by CDK1-cyclin B1 initiates its Cdc20-dependent destruction during mitotic arrest.* EMBO J, 2010. **29**(14): p. 2407-2420.

14. Huang, N.-J., et al., *The Trim39 ubiquitin ligase inhibits APC/CCdh1-mediated degradation of the Bax activator MOAP-1*, in *Journal of Cell Biology*2012. p. 361-367.

15. Garedew, A., C. Andreassi, and S. Moncada, *Mitochondrial Dynamics, Biogenesis, and Function Are Coordinated with the Cell Cycle by APC/CCDH1.* Cell Metabolism, 2012. **15**(4): p. 466-479.

16. Cho, H.J., et al., *Degradation of Human RAP80 is Cell Cycle Regulated by Cdc20 and Cdh1 Ubiquitin Ligases*, in *Molecular Cancer Research*2012. p. 615-625.

17. Liu, J., et al., *Phosphorylation of Mps1 by BRAFV600E prevents Mps1 degradation and contributes to chromosome instability in melanoma.* Oncogene, 2012.

18. Cui, Y., et al., *Degradation of the Human Mitotic Checkpoint Kinase Mps1 Is Cell Cycle-regulated by APC-cCdc20 and APC-cCdh1 Ubiquitin Ligases.* Journal of Biological Chemistry, 2010. **285**(43): p. 32988-32998.

19. von Klitzing, C., et al., *APC/C<sup>Cdh1</sup>-Mediated Degradation of the F-Box Protein NIPA Is Regulated by Its Association with Skp1.* PLoS ONE, 2012. **6**(12): p. e28998.

20. Takahashi, A., et al., *DNA Damage Signaling Triggers Degradation of Histone Methyltransferases through APC/CCdh1 in Senescent Cells.* Molecular Cell, 2012. **45**(1): p. 123-131.

21. Wang, R., et al., *Cdc20 mediates D-box-dependent degradation of Sp100.* Biochemical and Biophysical Research Communications, 2011. **415**(4): p. 702-706.

22. Kang, J., et al., *Dynamic Regulation of Oct1 during Mitosis by Phosphorylation and Ubiquitination.* PLoS ONE, 2011. **6**(8): p. e23872.
